# Prevalence and risk factors of the most common multimorbidity among Canadian adults

**DOI:** 10.1371/journal.pone.0317688

**Published:** 2025-01-22

**Authors:** Obed Mortey, Gerald Mugford, Kris Aubrey-Bassler, Hensley H. Mariathas, Ugochukwu Odimba, Zhiwei Gao

**Affiliations:** 1 Faculty of Medicine, Division of Population Health and Applied Health Sciences, Clinical Epidemiology Unit, Memorial University, St. John’s, Newfoundland and Labrador, Canada; 2 Factableulty of Medicine, Discipline of Family Medicine, Memorial University, St. John’s, Newfoundland and Labrador, Canada; Universidade Federal de Pelotas, BRAZIL

## Abstract

**Background:**

The number of persons living with multimorbidity–defined as the co-occurrence of at least two chronic conditions in the same individual–is growing globally, especially in developed countries. Traditionally, this increase has been attributed to a growing aging population, sedentary lifestyle, obesity, low socioeconomic status, and individual genetic susceptibility.

**Objective:**

To investigate the prevalence and associated risk factors of the most common multimorbidity (MCM) among Canadian middle-aged and older adults.

**Method:**

Relevant data on all 30,097 middle-aged and older Canadian adults (aged 45 to 85 years) from the Canadian Longitudinal Study on Aging were used for this study. To identify the specific sociodemographic risk factors associated with the MCM, we used survey-specific logistic regression.

**Findings:**

Overall, co-occurrence of osteoarthritis and hypertension was identified as the MCM among Canadian adults aged 45+ with an estimated prevalence of 16.5%. The results from multivariate analysis showed that seven factors were significantly associated with increased odds of the MCM, which included increasing age, being retired from work (retired vs not retired), poorer rating of perceived health, (very good, good, poor vs excellent), increasing problems with sleep quality (satisfied, dissatisfied vs neutral), and abnormal body-mass index (underweight, overweight, obese vs normal). Also, residents in other urban centres had significantly lower odds than those in urban core. Persons living in Atlantic Canada, Ontario and Quebec were at increased odds of having the MCM compared to those in British Columbia. The odds of the MCM associated with increasing age was significantly higher among Females (OR = 1.12, 95% CI = 1.11–1.13) than Males (OR = 1.08, 95% CI = 1.07–1.10).

**Conclusion:**

Multimorbidity is a common feature among Canadian adults. The identification of the most prevalent patterns and associated risk factors in this study provides fresh insights into the etiology, progression, and possible prevention of the MCM among Canadian adults.

## Introduction

The prevalence of chronic health conditions has been on the rise in recent times [1,2] leaving many people dependant on long-term management. Over the last few decades, the presence of more than one chronic health condition in a single individual–a phenomenon known as multimorbidity–has become common in our society and in primary care [3]. Multimorbidity is associated with significant increases in mortality, poor quality of life, and reduced functional status [4]. This has presented major challenges to healthcare systems globally and is considered a public health concern due to its association with the aging population, frailty, healthcare, and social care support systems [5,6].

In 2021, the global prevalence of multimorbidity was estimated at 37.2%, and was significantly higher in North America (43.1%) [7]. Several recent studies have estimated the prevalence in Canada to be 10–25% [8–11]. Discrepancy in the estimated prevalence may be due to differences in classification systems used to identify chronic health conditions, including the choice of conditions, and variations in study population [12]. This variation in prevalence estimates makes it difficult to compare the results from different studies and accurately quantify the specific burden of the most common multimorbidity that affect majority of the Canadian adult population. To address this challenge, there is the need for a more consistent approach to assessing multimorbidity. Prevalence studies of the most common multimorbidity, which employ data from a nationally represented sample, will serve as valuable sources of evidence that could be used to inform policymakers and public health experts in their decisions to develop and implement policies that focus on the allocation of resources in the management, treatment, and prevention of multimorbidity.

Multimorbidity patterns commonly identified include cardiovascular diseases, musculoskeletal disorders, metabolic disorders, and mental health disorders [13,14]. Due to the different sociodemographic factors and disease patterns, the prevalence and patterns of multimorbidity among Canadian adults may be different from those of other countries [15]. Although previous studies have investigated the burden of multimorbidity in Canada [8–11], the focus has been on describing the trend in terms of the number and disease patterns of the multiple chronic conditions among the Canadian population.

Therefore, the current study will focus on identifying the most common multimorbidity (MCM), and associated risk factors in a sample of middle-aged and older Canadians using data from the Canadian Longitudinal Study on Aging (CLSA). The CLSA is a national research initiative designed to explore the aging process and the factors that influence healthy aging. The study collects extensive data on health, lifestyle, social, and economic factors, which allow for a holistic understanding of aging and the factors that influence it. Particularly, the CLSA provides a robust and valuable dataset that can be used to explore the health dynamics and burden of multimorbidity among Canadian adults.

We believe that the outcome of this investigation will: a) help in the understanding of how chronic conditions coexist in middle-aged and older Canadians; b) inform public health experts on the necessary preventive measures needed in the growing fight against multimorbidity by targeting those modifiable factors identified by this study; c) help increase awareness of multimorbidity in Canada; and d) influence the creation and implementation of health policies that target the training and recruitment of specialized health professionals with expertise in the management of multimorbidity.

## Method

### Ethical approval

This CLSA project received ethics approval at two levels. Consent to participate was obtained for all participants under the CLSA harmonized multi-university ethics process approved by the Hamilton Integrated Research Ethics Board (HiREB), Hamilton Health Sciences/McMaster University. Simon Fraser University (SFU) was a participating institution in the CLSA data collection, and the SFU Office of Research Services Ethics Committee reviewed all consent material prior to data collection (SFU ORS #2018s0139). Ethics approval for the current study was obtained from Memorial University Health Research Ethics Board (HREB # 2022.115).

### Study population

The CLSA–consisting of a stratified random sample of over 50,000 community-dwelling Canadian men and women from age 45 to 85 –is one of the largest and most comprehensive national research platforms which examines many aspects of health and aging [16]. Participants were excluded from the study based on the following criteria: 1) inability to communicate in English or French; 2) cognitive impairment at the time of contact; 3) residents of the three territories; 4) full-time member of the Canadian Armed Forces; 5) resident in a long-term institution at the time of recruitment; and 6) living on Federal First Nations reserves or other First Nations settlements. All participants provided written consent for participation in the study. Data on a wide range of variables including sociodemographic and economic factors, lifestyle choices and behaviours, physical measurements, and biospecimens (blood and urine) were collected at the time of recruitment (baseline). According to the official documentation of the CLSA, the sampling plan used was nationally representative of Canadian adults >45 years of age for both urban and rural communities in Canada, and detailed descriptions of the design features of the CLSA have previously been published [16–18].

The CLSA is made up of two complementary cohorts: the Tracking cohort, which consists of 21,241 people who were questioned over the phone, and the Comprehensive cohort, which consists of 30,097 people who provided baseline data via an in-person home interview as well as other questionnaires, tests, physical measurements, and biospecimens (blood and urine) collected at the data collection sites [19].

In this study, we used baseline (cross-sectional) data from the CLSA’s comprehensive cohort which were collected between May 2012 and May 2015. The data module used was the questionnaire dataset, which contained participant information on physical assessments, blood biomarkers, sociodemographic and lifestyle behavioral factors.

### Definition of multimorbidity

Multimorbidity was defined as a positive response to the question, “Has a doctor ever told you that you have ___?” for any two of the following thirty chronic conditions.

The thirty chronic conditions were all self-reported physician-diagnosed conditions and were based on commonly reported chronic conditions in systematic reviews and multimorbidity studies in Canada [20–22]: Heart disease (including congestive heart failure), Peripheral vascular disease, Alzheimer’s disease, Multiple sclerosis, Epilepsy, Migraine headache, Stomach & intestinal ulcer, Bowel disorder, Bowel incontinence, Urinary incontinence, Macular degeneration, Cancer, Anxiety, Clinical depression, Backpain, Kidney disease, Rheumatoid arthritis, High blood pressure, Stroke, Asthma, Osteoporosis, Parkinson’s disease, Chronic obstructive pulmonary disease, Glaucoma, Diabetes, Osteoarthritis (hand, hip, or knee), Hyperthyroidism, Hypothyroidism, Angina (chest pain), and Blocked arteries (atherosclerosis). Anxiety and Clinical depression were grouped together as one chronic condition in our analysis.

### Predictor variables

The following sociodemographic and economic factors, lifestyle behaviours, and physical assessment variables from the CLSA questionnaire dataset were recoded and included: age as a continuous variable, self-reported sex (male, and female), education level (less than secondary education, secondary/some post-secondary education, and post-secondary education), marital status (single/never married, married/common-law relationship, and widowed/divorced/separated), urban-rural settlement (rural, urban core, and other urban centres), total annual household income (less than $50,000, $50,000 to less than $100,000, $100,000 to less than $150,000, and $150,000 or more), body-mass index (BMI) defined using International Standard (underweight:<18.5kg/m^2^, normal:18.5–24.9kg/m^2^, overweight:25–29.9kg/m^2^, and obese:≥30kg/m^2^), self-reported sleep quality (very satisfied/satisfied recoded as satisfied; neutral; and dissatisfied/very dissatisfied recoded as dissatisfied), self-reported general health (excellent, very good, good, and poor), homeownership (own, rent, and others), retirement status (completely retired/partly retired, and not retired), self-reported drinking status in the past twelve months (regular: at least once a month, occasional: less than once a month, and never), self-reported physical activity in the past seven days (never, seldom, and sometimes/often), self-reported smoking status in the past twelve months (current, never, and former), province at recruitment (Prairies, British Columbia, Eastern provinces, and Ontario/Quebec), and self-reported cultural/racial background (white, black, and others).

### Statistical analysis

We summarized the characteristics of the study population using the mean, standard error of mean, frequency, and percentage for the continuous and categorical variables, as appropriate.

For the regression analyses, the MCM was our outcome variable. Participants who responded “Yes” to the question, “Has a doctor ever told you that you have ___?”, for both osteoarthritis and high blood pressure were coded as 1. Those who responded “No” for both conditions were coded as 0. The MCM and associated risk factors were identified by survey-specific statistical procedures, which allowed us to account for sampling weights and complex survey design of the CLSA including stratification and clustering [23]. Sampling weights were applied for all statistical analyses: inflation weights for all descriptive analyses, and analytic weights for the regression analyses. Purposeful selection method was used to build our final model. All clinically important variables and variables which were significant at 0.20 level in the univariate analysis were included in an initial multivariate model. Only clinically important variables and the variables which were significant at p<0.05 remained in the final model. We explored the effect of the interaction between age and sex on multimorbidity. All analyses were performed using SAS software version 9.4 (SAS Institute Inc, Cary, NC).

## Results

Table 1 displays the weighted results of the characteristics of our study population. The proportion of females was 52.4%, and the average age of participants was 59.7 years. Most participants were highly educated with more than 60% having a post secondary education. A sizable proportion were in a marital or common-law relationship, but only a few were single or never married. Furthermore, 44.8% considered themselves either completely or partly retired, and more than 89% of study participants were concentrated in the urban core regions of Canada. The results also show that majority of the population were homeowners, and approximately 62.6% of households earn less than $100,000 annually with less than one-fifth making $150,000 or more.

**Table 1:** Characteristics of study population (weighted sample size, n = 3,812,085)^a^.

Characteristic	Category	Percentage %	95% CI
Age, mean ± S.E.M		59.7 ± 0.12	(59.5–60.0)
Sex	Females	52.4	(51.3–53.5)
	Males	47.6	(46.5–48.7)
Education level	Less than secondary education	17.4	(16.1–18.6)
	Secondary/some post-secondary education	20.6	(19.8–21.4)
	Post-secondary education	62.1	(60.9–63.3)
Marital status	Single/never married	8.7	(8.1–9.3)
	Married/common-law partnership	74.1	(73.2–75.0)
	Widowed/divorced/separated	17.2	(16.4–17.9)
Retirement status	Retired (completely/partly)	44.8	(43.7–45.9)
	Not retired	55.2	(54.1–56.3)
Urban-rural settlement	Rural	5.2	(4.8–5.7)
	Urban core	89.3	(88.7–89.9)
	Other urban centres	5.5	(5.0–6.0)
Total annual household income	0 –less than $50,000	29.4	(28.3–30.6)
	$50,000 –less than $100,000	33.2	(32.2–34.3)
	$100,000 –less than $150,000	20.0	(19.2–20.9)
	≥ $150,000	17.3	(16.5–18.1)
Body-mass index	Underweight	0.8	(0.6–1.1)
	Normal	29.4	(28.4–30.4)
	Overweight	39.0	(37.9–40.1)
	Obese	30.8	(29.7–31.8)
Self-reported sleep quality	Satisfied	57.9	(56.8–59.0)
	Neutral	15.3	(14.5–16.1)
	Dissatisfied	26.8	(25.8–27.8)
Self-reported general health	Excellent	18.0	(17.2–18.8)
	Very good	39.5	(38.4–40.6)
	Good	32.3	(31.3–33.4)
	Poor	10.2	(9.5–10.9)
Homeownership	Own	82.2	(81.4–83.1)
	Rent	16.9	(16.0–17.8)
	Others	0.9	(0.6–1.1)
Drinking habit	Regular	72.6	(71.6–73.7)
	Occasional	13.2	(12.4–14.0)
	Never	14.2	(13.3–15.0)
Physical activity	Never	65.7	(64.6–66.7)
	Seldom	14.4	(13.6–15.3)
	Sometimes/often	19.9	(19.0–20.7)
Smoking status	Current	12.1	(11.3–12.9)
	Never	43.4	(42.4–44.5)
	Former	44.4	(43.3–45.6)
Province at recruitment	AB, MN	17.6	(17.1–18.2)
	BC	28.3	(27.5–29.1)
	NL, NS	5.8	(5.7–6.0)
	ON, QB	48.3	(47.5–49.1)
Cultural background/race	White	92.3	(91.7–93.0)
	Black	1.2	(1.0–1.5)
	Others	6.4	(5.9–7.0)
Major chronic conditions and most common multimorbidity	OA-HBP	16.5*	(15.4–17.5)
	HBP	32.8	(31.7–33.8)
	BCKP	28.3	(27.3–29.3)
	OA	24.3	(23.4–25.3)
	DIA	17.4	(16.5–18.2)

MN: Manitoba; AB: Alberta; NS: Nova Scotia; NL: Newfoundland and Labrador; ON: Ontario; QB: Quebec; BC: British Columbia; S.E.M: standard error of the mean; OA: Osteoarthritis; HBP: High blood pressure; BCKP: Backpain disorder; DIA: Diabetes; CI: Confidence interval.

*: Prevalence rate of the most common multimorbidity.

^a^: The original unweighted sample size, n = 30,097.

According to Table 1 approximately 90% of participants self-rated their current health as being at least good. However, almost 27% of the population were not satisfied with the quality of sleep they were getting at night. More than 65% of the population did not engage in any physical activity in the past seven days, and the proportion of overweight and obese participants, as determined by standard BMI values, was high (approximately 70%). A significant number of subjects were classified as regular consumers of alcohol, and more than 56% of the population had a smoking history (i.e., either former or current smoker).

The eastern provinces (i.e., Newfoundland and Labrador, and Nova Scotia) accounted for the lowest proportion of recruits for this study (about 5.8%), and individuals of white ancestry were higher than any other race. The MCM was osteoarthritis-high blood pressure with a prevalence of 16.5% among middle-aged and older Canadians.

The results of the univariate analysis are summarized in Table 2. S1 Table is the supplementary information for Table 2. Age, sex, education level, marital status, retirement status, urban-rural settlement, total annual household income, BMI, self-rated sleep quality and general health, homeownership, drinking habit, physical activity, province at recruitment, cultural background, and smoking status were significantly associated with the odds of the MCM.

**Table 2:** Univariate analysis of sociodemographic factors and the MCM among Canadian adults (weighted sample size, n = 18564.89)^a^.

Variable	Category	OR (95% CI)	P-value
Age		1.10 (1.10–1.11)	<0.0001
Sex	Females	1.53 (1.37–1.71)	<0.0001
	Males	1	
Education level	Less than secondary education	3.07 (2.56–3.68)	<0.0001
	Secondary/some post-secondary education	1.42 (1.27–1.58)	<0.0001
	Post-secondary education	1	
Marital status	Single/never married	0.95 (0.73–1.25)	0.7364
	Widowed/divorced/separated	2.34 (2.08–2.63)	<0.0001
	Married/common-law partnership	1	
Retirement status	Retired (completely/partly)	4.85 (4.28–5.48)	<0.0001
	Not retired	1	
Urban-rural settlement	Rural	0.74 (0.62–0.88)	0.0009
	Other urban centres	0.70 (0.56–0.89)	0.0027
	Urban core	1	
Total annual household income	0 –less than $50,000	5.19 (4.34–6.20)	<0.0001
	$50,000 –less than $100,000	2.78 (2.33–3.32)	<0.0001
	$100,000 –less than $150,000	1.43 (1.18–1.75)	<0.0001
	≥ $150,000	1	
Body-mass index	Underweight	0.46 (0.19–1.09)	0.0775
	Overweight	2.03 (1.76–2.35)	<0.0001
	Obese	6.23 (5.39–7.20)	<0.0001
	Normal	1	
Self-reported sleep quality	Satisfied	1.10 (0.95–1.28)	0.2007
	Dissatisfied	1.44 (1.22–1.69)	<0.0001
	Neutral	1	
Self-reported general health	Very good	2.04 (1.70–2.46)	<0.0001
	Good	4.32 (3.57–5.23)	<0.0001
	Poor	8.69 (6.97–10.84)	<0.0001
	Excellent	1	
Homeownership	Own	0.50 (0.29–0.87)	0.0149
	Rent	0.89 (0.51–1.57)	0.6880
	Others	1	
Drinking habit	Regular	0.61 (0.52–0.72)	<0.0001
	Occasional	1.14 (0.93–1.41)	0.2117
	Never	1	
Physical activity	Never	2.32 (2.01–2.66)	<0.0001
	Seldom	1.12 (0.91–1.36)	0.2902
	Sometimes/Often	1	
Smoking status	Current	0.54 (0.44–0.67)	<0.0001
	Never	0.67 (0.60–0.75)	<0.0001
	Former	1	
Province at recruitment	AB, MN	1.18 (0.10–1.39)	0.0561
	NL, NS	1.36 (1.15–1.61)	0.0004
	ON, QB	1.30 (1.13–1.50)	0.0004
	BC	1	
Cultural background/race	White	1.50 (1.15–1.97)	0.0030
	Black	1.90 (1.05–3.45)	0.0353
	Others	1	

MN: Manitoba; AB: Alberta; NS: Nova Scotia; NL: Newfoundland and Labrador; ON: Ontario; QB: Quebec; BC: British Columbia; OR: Odds ratio; CI: Confidence interval.

^a^: The original unweighted sample size, n = 30,097.

As seen from Table 2, while the likelihood of having the MCM increases with age by about 10%, the association with sex is significantly higher among females compared to males. Moreover, participants with less than secondary education were about three times more likely than those with post-secondary education of developing the two conditions. A similar association is seen with the population of retirees (either partly or completely retired) versus non-retired (Retired vs Not Retired; OR: 4.85, CI = (4.28–5.48)). The results further show that an economic indicator, such as total annual income, is significantly associated with multimorbidity. Compared with the most affluent population, those who make the least annual income have the highest likelihood of having multimorbidity. The relationship between physical activity and multimorbidity in Table 2 indicates that those who reported to have never engaged in any physical activity for the past seven days were about 2.32 times more likely of having the MCM compared with their active counterparts.

The multivariate analysis in Table 3 showed a significant interaction between age and sex (p<0.001). S2 Table is the supplementary data for Table 3. S3 Table provides the main effect without the interaction term for the final model. The odds of the MCM associated with increasing age was significantly different between Females and Males: it was significantly higher in Females (OR = 1.12, 95% CI = 1.11–1.13) than Males (OR = 1.08, 95% CI = 1.07–1.10).

**Table 3:** Multivariate analysis of risk factors of the MCM among Canadian adults (weighted sample size, n = 18392.76)^a^.

Variable name	Category	OR (95% CI)	P-value
Age *	Female	1.12 (1.11–1.13)	<0.0001
	Male	1.08 (1.07–1.10)	<0.0001
Retirement status	Retired	1.43 (1.19–1.71)	<0.0001
	Not Retired	1	
Self-reported general health	Very good	1.97 (1.61–2.42)	<0.0001
	Good	3.65 (2.94–4.54)	<0.0001
	Poor	6.93 (5.37–8.94)	<0.0001
	Excellent	1	
Self-reported sleep quality	Satisfied	1.11 (0.93–1.33)	0.2501
	Dissatisfied	1.33 (1.10–1.61)	0.0038
	Neutral	1	
Body-mass index	Underweight	0.23 (0.09–0.61)	0.0030
	Overweight	2.09 (1.77–2.45)	<0.0001
	Obese	6.11 (5.16–7.24)	<0.0001
	Normal	1	
Urban-rural settlement	Rural	0.87 (0.70–1.08)	0.1915
	Other urban centres	0.69 (0.53–0.89)	0.0046
	Urban core	1	
Province at recruitment	AB, MN	1.19 (0.98–1.45)	0.0746
	NL, NS	1.35 (1.10–1.65)	0.0041
	ON, QB	1.26 (1.06–1.50)	0.0107
	BC	1	

MN: Manitoba; AB: Alberta; NS: Nova Scotia; NL: Newfoundland and Labrador; ON: Ontario; QB: Quebec; BC: British Columbia; OR: Odds ratio; CI: Confidence interval.

*: Interaction between age and self-reported sex (p<0.0001).

^a^: The original unweighted sample size, n = 30,097.

The participants who reported feeling unhealthy were at increased odds of multimorbidity compared to those who reported feeling healthier. The odds for those who reported feeling poor about their health was nearly twice as high as those who reported feeling good. Furthermore, a decrease in BMI tended to be associated with a reduced likelihood of multimorbidity. Also, retirement from work conferred a higher likelihood of multimorbidity on participants. Participants reporting dissatisfaction with sleep quality were 1.33 times more likely of having the MCM than those neutral respondents. Urban-rural settlement was significantly associated with the risk of MCM. In comparison with participants living in urban core areas, participants living in rural settlements were not significantly associated with increased odds of having the MCM, however, those living in other urban centres were significantly associated with lower odds of the MCM (OR = 0.69, 95% CI = 0.53–0.89, p<0.01). Living in Atlantic Canada (i.e., Newfoundland and Labrador, and Nova Scotia) was also significantly associated with increased likelihood of the MCM than those in British Columbia.

## Discussion

This study found that the co-occurrence of osteoarthritis and high blood pressure was the MCM among middle-aged and older Canadian adults. The estimated prevalence was found to be 16.5%. A total of seven sociodemographic factors were significantly associated with the MCM: age, retirement status, self-reported general health, self-reported sleep quality, body-mass index, urban-rural settlement, and province at recruitment. The odds of the MCM associated with increasing age was significantly higher in Females than in Males.

Given that multimorbidity is a combination of chronic conditions, the identification of osteoarthritis and high blood pressure as the MCM among Canadian adults merits a brief overview of these conditions. According to Coppola et al., osteoarthritis is a bone and joint condition and is characterized by progressive degradation of articular cartilage and remodelling of the underlying bone due to active response of chondrocytes [24]. High blood pressure is a common condition of arteries, and it is characterized by consistently elevated blood pressure against the artery walls. Although osteoarthritis and high blood pressure are two distinct diseases and the mechanism of pathogenesis of the two diseases is not clear, it is generally believed that many sociodemographic factors and genetic predisposition are the driving force behind development of the two diseases [25]. The identified sociodemographic risk factors of the co-occurrence of osteoarthritis and hypertension by this study shed light on the etiology of the two diseases.

Our results are consistent with a recent systematic review, which reported that the overall odds of having osteoarthritis was significantly increased in the people with hypertension compared to the normotensive ones (OR = 1.60, 95% CI = 1.33–1.94) [26]. Moreover, the association was significantly stronger in women when adjusted for BMI [26], a finding which harmonizes well with the results of this study.

This study reported that increasing age was significantly associated with greater odds of the MCM, and the association was significantly higher in Females than in Males (OR = 1.12 vs. OR = 1.08, p<0.001).

This may be partly explained by the fact that women are, in general, more health conscious and tend to visit healthcare providers more often and are therefore more likely to be diagnosed of hypertension than men as shown in some studies [27,28]. In Canada, hypertension has been found to be more prevalent in women than men [27].

Another explanation for the observed significant difference may be due to the role played by sex hormones. Since estrogens play a crucial role in cartilage protection, inflammation modulation, and bone metabolism promotion, hormonal fluctuations in females during menstrual cycles, and most notably postmenopausal changes, significantly increase disease incidence in the mid-forties age range [29,30].

Furthermore, sex-specific differences in joint alignment have been found to increase the incidence of osteoarthritis in females. Females generally exhibit higher quadriceps angle, lower arch height index, and a broader range of internal and external rotation in the hip joint, resulting in prolonged stress on the hip and knee joints over time [31]. Additionally, weight gain and the prolonged hormonal fluctuation in pregnancy weakens joints, particularly the lower joints and spine increasing the risk of osteoarthritis in females [32].

On the other hand aging is a gradual, continuous process of biological, physiological and psychological changes across all organ systems, and it has long been established as a common risk factor for many chronic diseases [33–39], including osteoarthritis and high blood pressure [24,40]. Many studies have shown that the most common risk factors for osteoarthritis are aging and obesity [41].

Telomere attrition is a feature of normal aging and has been found to be far greater in individuals with chronic diseases such as cardiovascular disease and diabetes [42,43]. Since high blood pressure is a precursor to many cardiovascular diseases, telomere attrition may also be involved in the shared pathway of disease development. Aside telomere attrition, there are many other potential mechanisms at cellular level that link aging with increased risk of multimorbidity.

Cellular senescence, for example, is a state of irreversible cell cycle arrest which occurs in response to cellular stressors such as DNA damage, and oxidative stress [37]. Cells enter senescence when DNA damage repairs are no longer effectively achieved and are subsequently programmed to die by apoptosis. New cells are eventually generated to replace damaged ones. Senescence is beneficial to cells as it maintains genomic stability and protects against the development of tumours. Chronic conditions of accelerated aging have been found to be associated with enhanced cellular senescence (possibly due to oxidative stress pathway), which–unlike apoptotic cells–remain metabolically active and may induce senescence in other neighbouring cells via senescence-associated secretory phenotype (SASP). SASP produces proinflammatory agents which mediate the development of complex chronic conditions such as chronic obstructive pulmonary disease (COPD), and cardiovascular diseases [37]. Senescent cells have been found to accumulate in conditions such as diabetes and cardiovascular diseases including hypertension [44]. Enhanced cellular senescence associated with chronic conditions and multimorbidity suggest a possible causal relationship which needs further exploration. More research is needed to establish a firm association between cellular senescence and the development of multimorbidity, especially with specific combinations such as osteoarthritis and high blood pressure.

A study on mice demonstrated that mice with T cells that are deficient in mitochondrial DNA-stabilizing protein have features associated with abnormal neurological, metabolic, muscular and cardiovascular functions and that these changes produce similar effects to inflammaging [45]. The study suggested that mitochondrial dysfunction can be used to predict multimorbidity via an important mediating factor (i.e., mitochondrial transcription factor A) which has been found to be associated with inflammaging.

Stem cells are responsible for replacing old worn-out and damaged cells with new and better ones. As cells age there is increased difficulty to function effectively resulting in impaired cell replacement and recovery [44]. Thus, stem cell exhaustion is a state of impaired stem cell function due to factors such as aging. Stem cell exhaustion is closely linked to cellular senescence, and as such may have synergistic effect which may help explain the progressive nature of multimorbidity especially among the aged.

These foregoing factors may help explain the significant sex/gender differences in the association between age and risk of the MCM.

Moving away from age and gender, participants who were partly or completely retired were at increased odds of developing the MCM compared to those in the workforce (OR = 1.43, 95% CI = 1.19–1.71, p<0.001) even after controlling for age. A similar result was reported in another cross-sectional study examining the prevalence of multiple chronic conditions among adults in Portugal attending primary care [46]. This current study suggests that the social engagements and daily routines at the workplace may offer protection against the development of the MCM identified (i.e., osteoarthritis and high blood pressure), and that individuals deprived of this psychosocial factor are more susceptible to developing the MCM. An alternative explanation would be that the development of multimorbidity leads to increased likelihood of retiring.

The association between self-reported general health and multimorbidity has been studied [47]. As expected, participants of the study with a poor/fair assessment of health were significantly more likely to be diagnosed with the MCM compared with those with excellent health reports (Table 3). Self-rated health has been reported in previous population-based studies to be significantly associated with multimorbidity and a good predictor of disease outcome [48,49]. These studies consistently show that persons who are diagnosed with more than one chronic condition, with at least one cardiovascular condition, are more likely to report poor self-rated health.

Our study showed participants’ sleep quality was significantly associated with risk of the MCM. There is evidence to suggest that insufficient sleep disrupts pathways related to cardio-metabolic, immune and inflammation [50], causing the release of stress hormones such as cortisol [51]. Furthermore, pain and discomfort associated with osteoarthritis, as well as the treatments and medications used for disease management, may lead to accumulation of sleep problems in patients with multimorbidity [52–54]. These may help explain the observed negative association between sleep satisfaction and the MCM. In a study investigating the association between sleep problems and multimorbidity patterns in older adults, participants with poor quality sleep were at greater odds of presenting with vascular-metabolic, cardiopulmonary, musculoskeletal, and coexisting conditions [55]. Other authors of a systematic review also found comparable results linking poor sleep quality and satisfaction with increasing odds of multimorbidity, including a previous study exploring sleep behaviors and multimorbidity occurrence in middle-aged and older adults using data from the CLSA [50,52].

Obese individuals are more at risk of developing hypertension and other related cardiovascular events [56] than those with normal BMI values. It is also important to note that increasing weight puts pressure on bones, particularly on the joints, and may cause or worsen conditions associated with the skeletal system (e.g., osteoarthritis). Our study suggests that obese individuals are approximately six times more likely to develop multimorbidity than those with normal BMI values (OR = 6.11, 95% CI = 5.16–7.24, p<0.0001). It is also worth noting that a lesser degree of excess weight significantly reduces the odds to less than half, suggesting that maintaining a healthy weight has a strong positive impact on reducing the risk of hypertension and osteoarthritis. In two systematic reviews and a meta-analysis to assess the impact of BMI on both knee and hip osteoarthritis, Jiang et al. found that a 5-unit increase in BMI was associated with a 35% increased risk of knee osteoarthritis (RR = 1.35, 95% CI = 1.21–1.51). This relationship was found, by the authors, to be significantly stronger in females than males (men, RR = 1.22; 95% CI = 1.19–1.25; women, RR = 1.38; 95% CI = 1.23–1.54; p = 0.04) [57,58]. A similar study exploring the risk factors of hypertension among Canadian adults aged 20 to 79 years found that being overweight or obese was one of the leading risk factors for hypertension among the Canadian population [59].

These findings, together with those of this study, underscore the need for increased public awareness of the health dangers posed by excessive weight gain. It should also be noted that although the underweight category seems to offer protection from multimorbidity when compared with those of normal weight from our study results (Table 3), such interpretations must be done cautiously especially due to the potential of survival bias, which could underestimate the health risk in this category and lead to wrong conclusions.

Urban-rural dwelling residence also affects the development of many health outcomes. Our study suggested that individuals in rural areas have relatively lower odds of having MCM compared with those in urban core areas. However, the association was not statistically significant (OR = 0.87, 95% CI = 0.70–1.08; p = 0.1915). In general, participants residing in other urban centres have significantly lower odds of MCM than those in urban core areas, and this should be a concern for policy makers and public health authorities since more than 89% of the middle-aged and older Canadian adults in this study live in these urban core regions. In a study examining the excess burden of osteoarthritis in the largest province of Canada (Ontario), Tarride et al. reported that almost 80% of study patients with osteoarthritis were living in urban areas, suggesting a greater concentration of the disease in urban areas of Canada [60]. Similarly, a population-based study conducted to estimate the prevalence and incidence of hypertension from 1995–2005 found that the percentage of patients diagnosed with high blood pressure was consistently higher in those living in urban areas [61]. The findings of Foguet-Boreu et al. in their study examining the impact of urban-rural settlement on multimorbidity in southern Europe, also suggest that living in rural areas, compared with urban areas, is significantly associated with a lower probability of multimorbidity [62] which supports the results of this study. Furthermore, a Scottish study describing the prevalence of multimorbidity among 41,545 hospitalized patients found that multimorbidity prevalence was 28.8% (95% CI = 28.1–29.5) in large urban versus 22.0% (95% CI = 20.9–23.3) in remote rural areas [63].

Factors influencing this observed disparity may include lifestyle choices, dietary habits, social support systems, and healthcare access. Rural dwellers typically engage in regular and more physical activities than urban dwellers due to their lifestyle, which can help maintain healthy weight and lower blood pressure. Also, the consumption of more traditional diets, whole foods, fruits and vegetables may contribute to the better overall health among rural dwellers. The strong social ties and support systems that exist in rural communities may further contribute to lower stress levels and better mental health, both of which are beneficial for promoting health. Moreover, the relatively reduced access to healthcare facilities in rural areas may lead to lower rates of diagnosis, as many remain undiagnosed, which can skew the data.

Participants residing in the eastern provinces of Canada (i.e., Newfoundland and Labrador, and Nova Scotia) at the time of recruitment were found to be significantly associated with increased odds of the MCM compared with those from British Columbia (OR = 1.35, 95% CI = 1.10–1.65, p = 0.0041). In a study exploring the regional variation in self-reported heart disease prevalence in Canada the authors found wide variations across provinces, with Nova Scotia and Newfoundland recording the highest [64]. The results of a large population survey, the Canadian Community Health Survey, also reported the proportion of high blood pressure among residents in eastern provinces was significantly higher than those in western provinces [65]. Similar regional variation in the prevalence of osteoarthritis was also reported by Statistics Canada, which showed that the prevalence in Atlantic provinces was higher than Canadian average, however, the prevalence in Alberta and BC was lower than Canadian average [66]. Similar studies also attribute increased prevalence of heart diseases and coexisting patterns to living in the Atlantic provinces [27]. This pattern is a replication of the east-to-west gradient of cardiometabolic health outcomes and represents a greater burden of chronic disease and multimorbidity on Atlantic Canada.

This observed regional variation in the prevalence of MCM is not fully understood. However, it could be attributed to many factors, such as lifestyle choices, socioeconomic factors, healthcare access, and demographic characteristics of residents in the eastern provinces. It is well established that a sedentary lifestyle and lower consumption of fruits and vegetables are associated with increased risk of both cardiovascular and musculoskeletal diseases. Also, economic challenges in some eastern provinces can lead to stress and limited access to healthy food options, which are essential for maintaining healthy lifestyle. This can exacerbate health issues including hypertension and osteoarthritis. Access to healthcare services is more limited in rural areas of eastern Canada, affecting the diagnosis and management of chronic health conditions, including hypertension and osteoarthritis. The recent pandemic further complicated healthcare access, leading to increased concerns about awareness and treatment of hypertension and other chronic health conditions. Finally, the population of eastern Canada may have a higher proportion of older people than other provinces, and thus may help explain the observed risk.

### Study strengths and limitations

This study was based on data drawn from a national, large-scale population study that employed standardized techniques and protocols to collect data to ensure accuracy and minimize bias. The integrity of the data collected is, therefore, high. Also, the results and interpretations can be generalized to the Canadian population [23]. This study included 30 self-reported physician-diagnosed chronic conditions, which, we believe, cover most common and important chronic conditions in middle-age and older Canadian adults based on the current literatures [20–22]. However, there are some limitations of this study. First, we recognize the issue of accuracy with self-reported medical conditions, which is subject to misclassification bias. If present, this could potentially impact the direction or validity of the results. However, nondifferential misclassification biases toward null. Although diagnosis was not objectively confirmed, it has been demonstrated that self-reported diagnosis of prevalent conditions are valid and reliable in epidemiological studies [67]. Second, our study is a cross-sectional study since we used the baseline information of the CLSA. Hence, we cannot draw any causal relationship. Finally, we are not oblivious to the possibility of reverse causal relationship between some risk factors identified and multimorbidity. The presence of multimorbidity can negatively impact the overall health and well-being of individuals. Although we demonstrate in our study that self-rated sleep deprivation and poor health, for example, are important risk factors associated with the development of osteoarthritis and high blood pressure, it is equally important to emphasize that these factors might as well be due to the presence of multimorbidity.

## Conclusion

Our study identified the co-occurrence of osteoarthritis and high blood pressure as the MCM among the Canadian adult population aged 45 to 85 years. The identified risk factors for the MCM provide insights into the etiology of MCM, which has implications for researchers, policy makers and public health authorities. Finally, the results of our study provide important information for future epidemiological and genetic-epidemiological studies of MCM in middle-aged and older Canadian adults.

## Supporting information

S1 Table Univariate analysis. (PDF)

S2 Table Multivariate analysis. (PDF)

S3 Table Multivariate analysis without interaction term. (PDF)
