# Correction: Prevalence and risk factors of the most common multimorbidity among Canadian adults

**DOI:** 10.1371/journal.pone.0321700

**Published:** 2025-04-08

**Authors:** Obed Mortey, Gerald Mugford, Kris Aubrey-Bassler, Hensley H. Mariathas, Ugochukwu Odimba, Zhiwei Gao

The phrase “an analysis of CLSA data” is missing from the title. The correct title is: Prevalence and risk factors of the most common multimorbidity among Canadian adults: an analysis of CLSA data. The correct citation is: Mortey O, Mugford G, Aubrey-Bassler K, Mariathas HH, Odimba U, Gao Z (2025) Prevalence and risk factors of the most common multimorbidity among Canadian adults: an analysis of CLSA data. PLoS ONE 20(1): e0317688. https://doi.org/10.1371/journal.pone.0317688.

The following information is missing from the funding statement: The opinions expressed in this manuscript are the authors’ own and do not reflect the views of the Canadian Longitudinal Study on Aging.

The Acknowledgments section is missing from the published article. The correct Acknowledgments section is as follows: This research was made possible using the data/biospecimens collected by the Canadian Longitudinal Study on Aging (CLSA). Funding for the Canadian Longitudinal Study on Aging (CLSA) is provided by the Government of Canada through the Canadian Institutes of Health Research (CIHR) under grant reference: LSA 94473 and the Canada Foundation for Innovation, as well as the following provinces, Newfoundland, Nova Scotia, Quebec, Ontario, Manitoba, Alberta, and British Columbia. This research has been conducted using the CLSA Baseline Comprehensive Dataset version 7.0, under Application ID 2206005. The CLSA is led by Drs. Parminder Raina, Christina Wolfson and Susan Kirkland.
