# Supplementary material for: Prevalence and risk factors of the most common multimorbidity among Canadian adults
Source: PLoS One. 2025 Jan 22;20(1):e0317688. doi: 10.1371/journal.pone.0317688 (PMC11753687; doi:10.1371/journal.pone.0317688)
Supplement: S1 Table — (PDF) [file pone.0317688.s001.pdf]

S1 Table. Univariate Analysis

Parameter	Category	Beta estimate	Standard error	P-value
Age		0.0977	0.00297	<0.0001
Sex	Females	0.4251	0.0566	<0.0001
	Males	0	-	-
Education level	Less than secondary education	1.1210	0.0930	<0.0001
	Secondary/some post-secondary education	0.3495	0.0565	<0.0001
	Post-secondary education	0	-	-
Marital status	Single/Never married	-0.0468	0.1389	0.7364
	Widowed/divorced/separated	0.8494	0.0594	<0.0001
	Married/Common-law partnership	0	-	-
Retirement status	Retired (completely/partly)	1.5779	0.0627	<0.0001
	Not retired	0	-	-
Urban-rural settlement	Rural	-0.3010	0.0908	0.0009
	Other urban centres	-0.3517	0.1174	0.0027
	Urban core	0	-	-
Total annual household income	0 – less than $50,000	1.6465	0.0910	<0.0001
	$50,000 – less than $100,000	1.0235	0.0905	<0.0001
	$100,000 – less than $150,000	0.3605	0.1007	<0.0001

	≥ $150,000	0	-	-
Body-mass index	Underweight	-0.7844	0.4443	0.0775
	Overweight	0.7094	0.0740	<0.0001
	Obese	1.8295	0.0740	<0.0001
	Normal	0	-	-
Self-reported sleep quality	Satisfied	0.0974	0.0761	0.2007
	Dissatisfied	0.3618	0.0819	<0.0001
	Neutral	0	-	-
Self-reported general health	Very good	0.7145	0.0948	<0.0001
	Good	1.4634	0.0973	<0.0001
	Poor	2.1625	0.1126	<0.0001
	Excellent	0	-	-
Homeownership	Own	-0.6889	0.2829	0.0149
	Rent	-0.1163	0.2896	0.6880
	Others	0	-	-
Drinking habit	Regular	-0.4956	0.0828	<0.0001
	Occasional	0.1349	0.1080	0.2117
	Never	0	-	-
Physical activity	Never	0.8393	0.0716	<0.0001
	Seldom	0.1085	0.1026	0.2902
	Sometimes/Often	0	-	-
Smoking status	Current	-0.6153	0.1112	<0.0001

	Never	-0.4012	0.0565	<0.0001
	Former	0	-	-
Province at recruitment	AB, MN	0.1634	0.0855	0.0561
	NL, NS	0.3069	0.0872	0.0004
	ON, QB	0.2629	0.0736	0.0004
	BC	0	-	-
Cultural background/race	White	0.4078	0.1372	0.0030
	Black	0.6407	0.3043	0.0353
	Others	0	-	-

Table shows data for univariate regression analysis of sociodemographic factors of the most common multimorbidity (i.e., osteoarthritis-high blood pressure) among a sample of middle-aged and older Canadian adults.

MN: Manitoba; AB: Alberta; NS: Nova Scotia; NL: Newfoundland and Labrador; ON: Ontario; QB: Quebec; BC: British Columbia; OR: Odds ratio; CI: Confidence interval.
