# Supplementary material for: Prevalence and risk factors of the most common multimorbidity among Canadian adults
Source: PLoS One. 2025 Jan 22;20(1):e0317688. doi: 10.1371/journal.pone.0317688 (PMC11753687; doi:10.1371/journal.pone.0317688)
Supplement: S2 Table — (PDF) [file pone.0317688.s002.pdf]

S2 Table. Multivariate Analysis

Parameter	Category	Beta estimate	Standard error	P-value
Age *	Female	0.1121	0.005460	<0.0001
	Male	0.08107	0.006097	<0.0001
Retirement status	Retired	0.3557	0.0911	<0.0001
	Not Retired	0	-	-
Self-reported general health	Very good	0.6792	0.1049	<0.0001
	Good	1.2949	0.1112	<0.0001
	Poor	1.9361	0.1300	<0.0001
	Excellent	0	-	-
Self-reported sleep quality	Satisfied	0.1055	0.0918	0.2501
	Dissatisfied	0.2825	0.0977	0.0038
	Neutral	0	-	-
Body-mass index	Underweight	-1.4696	0.4958	0.0030
	Overweight	0.7347	0.0826	<0.0001
	Obese	1.8100	0.0864	<0.0001
	Normal	0	-	-
	Rural	-0.1436	0.1099	0.1915

Urban-rural settlement	Other urban centres	-0.3721	0.1314	0.0046
	Urban core	0	-	-
Province at recruitment	AB, MN	0.1761	0.0987	0.0746
	NL, NS	0.2972	0.1035	0.0041
	ON, QB	0.2288	0.0897	0.0107
	BC	0	-	-

Table shows data for final regression model for risk factors of the most common multimorbidity (i.e., osteoarthritis-high blood pressure) among a sample of middle-aged and older Canadian adults.

MN: Manitoba; AB: Alberta; NS: Nova Scotia; NL: Newfoundland and Labrador; ON: Ontario; QB: Quebec; BC: British Columbia; OR: Odds ratio; CI: Confidence interval.

*: Interaction between age and self-reported sex ($p<0.0001$).
