# Supplementary material for: Prevalence and risk factors of the most common multimorbidity among Canadian adults
Source: PLoS One. 2025 Jan 22;20(1):e0317688. doi: 10.1371/journal.pone.0317688 (PMC11753687; doi:10.1371/journal.pone.0317688)
Supplement: S3 Table — (PDF) [file pone.0317688.s003.pdf]

S3 Table. Multivariate Analysis of Risk Factors without interaction term (weighted sample size, n= 18392.76)[a]

Variable name		OR (95% CI)	P-value
Age	Years	1.10 (1.09 – 1.11)	<0.0001
Sex	Female	1.66 (1.45 – 1.89)	<0.0001
	Male	1	
Retirement status	Retired	1.41 (1.18 – 1.68)	0.0002
	Not retired	1	
Self-reported general health	Very good	1.99 (1.62 – 2.44)	<0.0001
	Good	3.66 (2.95 – 4.55)	<0.0001
	Poor	6.92 (5.36 – 8.94)	<0.0001
	Excellent	1	
Self-reported sleep quality	Satisfied	1.10 (0.92 – 1.32)	0.2964
	Dissatisfied	1.31 (1.08 – 1.59)	0.0053
	Neutral	1	
Body-mass index	Underweight	0.25 (0.10 – 0.64)	0.0037
	Overweight	2.06 (1.76 – 2.42)	<0.0001
	Obese	6.05 (5.12 – 7.13)	<0.0001
	Normal	1	
Urban-rural settlement	Rural	0.86 (0.69 – 1.06)	0.1636
	Other urban centres	0.69 (0.53 – 0.90)	0.0052

	Urban core	1	
Province at recruitment	AB, MN	1.20 (0.99 – 1.45)	0.0663
	NL, NS	1.35 (1.10 – 1.65)	0.0041
	ON, QB	1.26 (1.06 – 1.50)	0.0095
	BC	1	

Table shows data for the final regression model without the interaction term for the risk factors of the most common multimorbidity among Canadian adults.

MN: Manitoba; AB: Alberta; NS: Nova Scotia; NL: Newfoundland and Labrador; ON: Ontario; QB: Quebec; BC: British Columbia; OR: Odds ratio; CI: Confidence interval.

[a]: The original unweighted sample size, n=30,097.
